# Decreased respiratory performance of children and adolescents with myelomeningocele who use a wheelchair – preliminary data

**DOI:** 10.1590/1414-431X20198671

**Published:** 2019-08-05

**Authors:** E.J. Martins, A.C. Gastaldi, G.B.Q. Davoli, M.M. Leonardi-Figueiredo, A.C. Mattiello-Sverzut

**Affiliations:** Departamento de Ciências da Saúde, Faculdade de Medicina de Ribeirão Preto, Universidade de São Paulo, Ribeirão Preto, SP, Brasil

**Keywords:** Spirometry, Lung function, Non-ambulatory, Strength, Spina bifida

## Abstract

Myelomeningocele (MMC) is a neural tube defect that often causes spinal cord injury at the thoracolumbar region, as well as sensory and motor paralysis in the lower limbs. This leads to continuous use of a wheelchair and, consequently, a sedentary lifestyle, predisposition to muscle weakness, cardiovascular and respiratory disorders, obesity, and structural alterations in the spine. We assessed the respiratory function and shoulder strength of MMC participants who were wheelchair-users and had no respiratory complaints and compared them to healthy children and adolescents. MMC (n=10) and healthy (n=25) participants of both genders with a mean age of 12.45 years (SD=2.1) were assessed for weight, height, respiratory performance, and isometric peak for shoulder flexors, extensors, abductors, and adductors, using an isokinetic dynamometer. Medullary lesion, functional levels, and abnormal curvatures of the spine were assessed for MMC participants. The level of spinal cord injury for the majority of the MMC participants was high lumbar and they had scoliosis. MMC showed lower values for forced vital capacity, forced expiratory volume at the first second, forced expiratory flow (25–75%), maximal voluntary ventilation, and isometric peak for shoulder flexors and adductors compared to healthy participants. This indicated a decreased vital capacity, respiratory muscle endurance, and shoulder muscle strength.

## Introduction

Myelomeningocele (MMC) is the most common and severe form of birth defect involving the neural tube. Scientific advances have increased the patients' longevity, while increasing their wheelchair dependence (1). Spinal cord injury at the sacral level occurs only in 20% of the children with MMC, allowing for greater possibility of independent ambulation. In most cases, the spinal cord injury is thoracolumbar and these children use wheelchairs for locomotion. Additionally, only 50% of patients with MMC with a high lumbar lesion continue to walk during adolescence (2). The continuous use of wheelchairs leads to a sedentary lifestyle and muscle skeletal weakness, which in turn increases the structural alterations of the spine such as, scoliosis and hyperkyphosis (3). Concomitantly, these factors contribute to obesity and cardiopulmonary diseases in adolescence and adulthood, impairing physical fitness (4). Aerobic activity has a high energy cost in this population, which leads to increased fatigue, discomfort, and consequently hypoactivity and sedentary behavior (5). Physical fitness has been widely researched and used to implement activities to promote physical conditioning (6).

Only a few studies have focused on the respiratory changes of the MMC population, which could be related to the poor symptomatology and clinical appeal. Although wheelchair-users with MMC represent the majority of the cases and present lower functional performance and greater tendency to develop comorbidities, most studies are composed of participants with domestic or community gait, who use sticks, crutches, walkers, or canes, and they are not exclusive wheelchair-users (participants that do not have any type of gait) (5,7).

Patel et al. (8) showed that patients with myelodysplasia had a reduced forced vital capacity (the percentage of predicted values for age was used for analysis), but this reduction was not related to the increased scoliosis observed. However, the authors did not describe the levels of spinal cord injury and the locomotion prognosis of the participants.

Even without respiratory complaints and/or symptoms, wheelchair dependency should be taken into consideration in this specific group of patients, considering the low intensity and/or low respiratory demand of daily life activities, which could be masking an important decrease of respiratory function and endurance.

We aimed to evaluate the respiratory function of MMC participants who had no respiratory complaints and were exclusive wheelchair-users and compare them to healthy children and adolescents. The comparison intended to demonstrate the gap between full and normal responses of respiratory function in healthy children, compared to the MMC participants who were strictly wheelchair-dependent. In addition, we investigated the strength of upper limbs and scoliosis to know if they can influence respiratory variables. Our hypotheses were: 1) respiratory variables and shoulder muscle strength are impaired in MMC, compared to healthy participants matched by age and gender, and 2) a negative correlation between respiratory function and scoliosis in wheelchair-users exists. Because patients with scoliosis show decreased thoracic cavity expansibility and restriction of pulmonary ventilation, and consequently limitation of exercise tolerance and decreased functional capacity (5), they have decreased general performance, leading to the vicious cycle of hypoactivity and sedentary life style in this population.

## Material and Methods

In this cross-sectional study, 35 participants between 10 and 16 years of age (mean=12.45 years; SD=2.1) were assigned to one of two groups: the MMC group (wheelchair-users, n=10; 6 girls and 4 boys) and the healthy participants group (n=25; 11 girls and 14 boys). MMC participants were recruited from the rehabilitation center at the Ribeirão Preto Medical School, University of São Paulo. Of the 44 patients with MMC who were being treated at this rehabilitation center only the non-ambulatory patients (n=20) were invited to participate in this study. Of these 20, only 18 patients met the inclusion criteria as follows: 1) radiological images showing thoracic, high lumbar, or low lumbar spinal cord injury; 2) between the ages of 6 and 18 years, and 3) use of wheelchair exclusively. The exclusion criteria were: a) presence of other morbidities and b) not understanding the evaluator commands. All MMC participants used a manual wheelchair. Of the 18 eligible patients, 8 refused to participate and/or could not complete the tasks. All patients used a ventriculoperitoneal valve to drain off cerebrospinal fluid, however, they did not show any clinical pyramidal signs. The healthy participants were recruited from public and/or private schools of Ribeirão Preto (Brazil) and surrounding towns. After collecting the anthropometric data of 516 healthy children and adolescents, 43 were invited to participate in the study. These participants were matched by age and gender to the MMC participants. All of the MMC participants matched at least two participants of the healthy group, but only 25 agreed to participate (11 girls and 14 boys). All parents and/or caregivers provided their written consent prior to the start of the study. This study was approved by the Research Ethics Committee of the Ribeirão Preto Medical School, University of São Paulo (Protocol number 371.508).

All participants were assessed for weight (kg) and height (cm). Wheelchair-users were weighed on a special scale (Welmy^®^ W200/5, Brazil) and their estimated height was obtained by arm span (wingspan or length between the middle finger of one hand to the other). The method is an adequate option for measuring the growth of children with MMC and has a good correlation with the conventional way of measuring height (9). The neurological levels of the lesion were obtained from medical records of the MMC participants and were given based on radiographic images, analyzed by their doctors, at the follow-up scheduled for each child and adolescent. The functional level was assessed using the protocol described by Hoffer et al. (10). The abnormal curvatures were classified as present or absent. Before the pulmonary function measurement, all participants answered questions about their health, including questions about dyspnea, weakness, and fatigue regardless of the situation (during activities of daily living or at the end of physical activity).

A spirometer (KoKo PTF System, version 4.11, 2007, nSpire Health Inc., Pulmonary Data Services, USA) was used to evaluate pulmonary function, following the protocol of the American Thoracic Society / European Respiratory Society (11). The measurements were obtained while the participant was sitting, wearing a nose clip, and breathing through a mouthpiece connected to a device, affixed between occluded teeth and lips to avoid air leaking. The variables evaluated in maneuvers of forced expiration were: forced vital capacity (FVC), forced expiratory volume at the first second (FEV_1_), FEV_1_/FVC ratio, forced expiratory flow between 25 and 75% of FVC (FEF 25–75), and maneuvers of maximal and fast breathing were analyzed using maximal voluntary ventilation (MVV).

In the absence of obstructive, restrictive, or mixed disorders, MVV shows the capacity of the respiratory muscles to generate volume throughout the effort. The higher the ventilatory capacity, the greater the lactate clearance and the better the exercise capacity.

We used at least 3 acceptable curves and the best obtained values. The data are reported as percentage of the expected values for gender, age, and height. A description of each variable is shown in Table 1. Values below 80% of the predicted values were considered abnormal.

**Table 1:** Spirometric variables.

Spirometric variables	Description
Forced vital capacity (FVC)	Maximum volume of air exhaled with maximum effort, from the point of maximum inspiration
Forced expiratory volume at the first second (FEV_1_)	Volume of air exhaled during the first second of expiration
FEV_1_/FVC	FEV_1_/FVC ratio
Forced expiratory flow (FEF)	Maximum air flow during the forced vital capacity maneuver
Forced expiratory flow (FEF) between 25 and 75% of FVC	Mean forced expiratory flow of a segment obtained during the FVC maneuver; i.e., between 25 and 75% of the FVC curve
Maximal voluntary ventilation (MVV)	Maximum volume of ventilated air in the period by repeated forced breathing maneuvers

The isokinetic evaluation was performed using an isokinetic dynamometer (Biodex Mult Joint System 4^®^, USA). The equipment was calibrated according to the manufacturer's instructions. After performing a warm-up in an arm cycle ergometer, without load for 3 min, the participants sat on the dynamometer chair with a back angle set at 90°, stabilized with belts on their chest, pelvis, and arms. Only the dominant limb was evaluated. For the shoulder measurements, we used the pediatric accessory Biodex^®^ (Biodex Mult Joint System 4^®^, USA). Immediately before data collection, the participants were familiarized with the equipment, by performing a total of 3 submaximal contractions for each movement (shoulder flexors, extensors, abductors, and adductors). For evaluation of the isometric peak for the shoulder flexors (iP SFL) and extensors (iP SEX), the mechanical axis of the dynamometer rotation was aligned with the greater tuberosity of the humerus, the handle remained in neutral position, the elbow in full extension, and the shoulder was positioned at a 90° flexion. For the evaluation of the isometric peak for the shoulder abductors (iP SAB) and adductors (iP SAD), the mechanical axis of the dynamometer rotation was aligned with the acromioclavicular joint, the handle remained in a neutral position, the elbow in full extension, and the shoulder was positioned at a 45° abduction.

During evaluation of the isometric peak, the participants performed 3 maximal contractions, sustained for 5 s, with intervals of 20 s between each of them and a 90 s rest between each movement. During the tests, the participants were verbally encouraged to use maximum force during contractions.

The results are reported as means±SD. Univariate normality was checked by the Shapiro-Wilk test, followed by Student's *t*-test for data with normal distribution, or Wilcoxon-Mann-Whitney test for data with non-normal distribution. Comparisons were made between the anthropometric data, spirometry variables (FVC, FEV_1_, FEV_1_/FVC, FEF, FEF 25–75%, and MVV) and shoulder muscle strength (iP SFL, iP SEX, iP SAB, and iP SAD), with wheelchair-users and healthy as the independent variables. Correlation analysis by Spearman coefficient was used to assess the correlation between abnormal curvature of the spine (scoliosis) and respiratory variables for the MMC. We used SPSS Statistics^®^ (version 20, USA) to process the data, with significance level set at 0.05.

## Results

Levels of spinal cord injury for the wheelchair-users were as follows: 36.4% thoracic level, 45.4% high lumbar level, and 18.3% lower lumbar level. Of the 10 wheelchair-users, 7 had scoliosis. Table 2 shows the anthropometric, respiratory function variables, and dynamometric strength variable (isometric peak-iP). Wheelchair-users showed significantly lower values for weight and height, compared to the healthy group (P≤0.05).

**Table 2:** Anthropometric, spirometric, and dynamometric variables for the participants.

	Wheelchair-users (n=10)	Healthy participants (n=25)
Age (years)	11.90 (10.13–13.68)	12.63 (11.65–13.62)
Weight (kg)^*^	37.65 (28.52–46.77)	53.25 (46.30–60.21)
Arm span/Height (cm)	140.70 (128.35–153.05)	154.72 (148.33–161.11)
FVC (%)^#^	74.60 (58.34–90.85)	104.20 (98.63–109.76
FEV_1_ (%)^#^	70.90 (51.60–90.19)	107.32 (100.00–114.63)
FEV_1_/FVC (%)	103.30 (91.27–115.32)	104.72 (102.22–107.21)
FEF25-75 (%)^*^	73.90 (43.71–104.08)	105.92 (94.82–117.01)
MVV (%)^#^	60.20 (36.85–83.54)	144.10 (120.45–167.82)
iP SFL (Nm)^#^	24.55 (8.10–60.87)	29.73 (10.33–67.20)
iP SEX (Nm)	33.32 (11.57–86.43)	36.81 (14.17–74.93)
iP SAB (Nm)	32.31 (9.30–76.00)	34.94 (15.60–84.87)
iP SAD (Nm)^#^	33.85 (12.53–98.63)	45.53 (18.03–96.53)

Data are reported as means and 95% confidence intervals (lower and upper bound). FVC: forced vital capacity; FEV_1_: forced expiratory volume at the first second; FEF25–75%: forced expiratory flow between 25 and 75% of FVC; MVV: maximal voluntary ventilation; iP SFL: isometric peak of the shoulder flexors; iP SEX: isometric peak of the shoulder extensors; iP SAB: isometric peak of the shoulder abductors; iP SAD: isometric peak of the shoulder adductors. *P≤0.05 and ^#^P≤0.01, wheelchair-users compared to healthy participants (arm span/height was not compared statistically between wheelchair-users and healthy participants). Student's *t*-test or Wilcoxon-Mann-Whitney test were used.

During forced expiratory maneuvers, wheelchair-users showed significantly lower FVC, FEV_1_, FEF 25–75% of FVC, compared to healthy controls (P≤0.05). Equivalent results were obtained for MVV, with a lower percentage for wheelchair-users, compared to the healthy group (P≤0.01) (Table 2). It is important to emphasize that the mean predicted values of MVV for wheelchair-users was 60.2%, while for the healthy group, it was 144.1%.

Analysis revealed decreased iP SFL and iP SAD for wheelchair-users, compared to the healthy group (P≤0.01).

Table 3 shows the correlation between scoliosis and respiratory function variables for the wheelchair-users, with a low negative correlation between scoliosis and FEF 25–75% (r=−0.40; P≤0.05) and a moderate negative correlation between scoliosis and FEV_1_ (r=−0.60; P≤0.01), FVC (r=−0.63; P≤0.01), and MVV (r=−0.58; P≤0.01).

**Table 3:** Correlation between scoliosis and spirometric variables measured in participants with myelomeningocele.

	FVC (r)	FEV_1_ (r)	FEV_1_/FVC (r)	FEF25-75 (r)	MVV (r)
Scoliosis	−0.63^#^	−0.60^#^	0.16	−0.40^*^	−0.58^#^

FVC: forced vital capacity; FEV_1_: forced expiratory volume at the first second; FEF25–75%: forced expiratory flow 25–75 between 25 and 75% of FVC; MVV: maximal voluntary ventilation; r: correlation analysis by Spearman coefficient (*P≤0.05 and ^#^P≤0.01).

## Discussion

MMC wheelchair-users presented decreased respiratory function, even though there were no respiratory complaints. Mean weight and height were lower among children with MMC, compared to healthy children. Nothing was found in the medical records that could justify this difference; no comorbidities were found.

The changes in morphology and function of the respiratory system in MMC have been observed by other authors, who have correlated them to changes in ventilation control, spinal deformities, respiratory muscle weakness, and low level of physical activity, which contribute to reduced pulmonary compliance and number of available alveoli, generating areas of hypoventilation (5).

These changes may limit complete pulmonary inflation, which is characteristic of restrictive respiratory disorders. Our patients showed FVC of 71%, which indicates slight restriction. The normal FEV_1_/FVC ratio indicated that our patients did not present obstructive disorders, however, the slight FEV 25–75% impairment may be related to the decrease in lung volume or some degree of peripheral obstruction.

It is worth noting the decrease in MVV (60% of predicted) for wheelchair-users, compared to the healthy group. As the participants in this study did not present obstructive disorder, which could interfere with these measurements, the decrease in MVV might be associated with a decrease in maximal ventilator performance, an indicator of decreased respiratory muscle endurance capacity.

The respiratory endurance evaluated through this dynamic maneuver (MVV) indicates the degree of ventilatory limitation. Their values can bring important inferences about the relationship of the respiratory system to exercise capacity, since it is better related to physical exertion than FEV_1_. Inadequate ventilation may compromise oxygen supply to the peripheral muscles, leading to impairment during physical exercises and daily living activities (12).

As far as we know, this evaluation has only been described by Sherman et al. (5), showing a decrease in MVV (68%) for MMC, but with a less pronounced difference, compared to the control group, which obtained 79% of MVV (6,8). It is interesting to note that the adduction strength, that involves muscles that are also breathing accessories, such as pectoralis major, is decreased, explaining why the MVV values of our subjects were lower than the ones described by Sherman (5).

Additionally, the low level of physical activity and low physical conditioning can also be causal factors for the respiratory limitation seen in MMC patients, as described by Sherman et al. (5), who attributed the lower values of MVV to the low level of physical activity in ambulatory MMC. The MMC participants had abnormal MVV values, indicating the inability of the respiratory muscle to offer resistance to fatigue or maintain a predetermined performance (endurance), in addition to the restrictive ventilatory disorder.

Among the deformities of the thoracic cavity, scoliosis is the most observed in MMC patients with thoracic or thoracolumbar lesion levels (13). In the present study, of the 10 patients, 45.4% had a high lumbar injury and 36.4% had a thoracic injury level, and scoliosis was present in 7. Patel et al. (8) assessed the relationship between spine deformity, lung capacity, and pressure ulcer occurrence, and found no relationship between lung function and Cobb angle progression. However, in the present study, we obtained moderate correlations between scoliosis and FVC, which may suggest some degree of association, i.e., scoliosis negatively influencing the respiratory function of the wheelchair-users with MMC. Moreover, studies have shown an increase in FVC and peak expiratory flow, after spinal corrective surgery in MMC (14).

De Groot et al. (1) identified the presence of high values of ventilatory equivalents of oxygen, carbon dioxide, and dyspnea in MMC, using cardiopulmonary exercise tests for the lower limbs, a finding that indicated the presence of ventilatory limitation in this group. Contrary to this finding, a study developed by our research group using cycloergometer for the upper limbs (15) did not find dyspnea and elevated values of the ventilatory equivalent of carbon dioxide in MMC wheelchair-users. However, we did report peripheral muscle fatigue and elevated values of ventilatory equivalent of oxygen. Thus, the decrease in respiratory muscle strength (7), restrictive ventilatory disorder, and low respiratory muscle endurance may also be related to sedentary lifestyle, low-intensity daily activities, and low demand on cardiorespiratory, muscular, and metabolic systems.

Therefore, it is important to note that, even in the absence of respiratory symptoms and complaints, respiratory changes may be present and progress throughout the life of MMC patients, mainly for wheelchair-users. These changes are very important for the evaluation and follow-up of these patients, suggesting the need for early planning and preventive interventions in respiratory and motor physical therapy to increase quality of life and prevent possible pulmonary complications in these patients, especially after surgery (16).

In the present study, wheelchair-users showed lower values for iP SFL and iP SAD, compared to the healthy group, as was expected. MMC participants were expected to show lower isometric muscle strength of the upper limbs, compared to their healthy counterparts. In 2008, Buffart et al. (17) used the handheld dynamometer to evaluate the isometric muscle strength of the abductors and elbow extensors of the adolescents and young adults with MMC. They concluded that 54% of non-ambulatory and 79% of ambulatory participants had subnormal muscle strength in at least one of the muscle groups. Schoenmakers et al. (18) observed lower isometric muscle strength of shoulder abductors and wrist extensors in children with spina bifida compared to reference values, using the strength manual test and a handheld dynamometer. It is suggested that the confinement to the wheelchair promotes a vicious cycle of inactivity and muscle disuse, negatively affecting the generation of muscle strength.

It is possible that, as also occurs in patients with neuromuscular diseases, patients with MMC could benefit from improved strength and respiratory endurance for locomotion and an earlier intervention, and therefore, prolonged training might be beneficial. In a recent systematic review, the authors discuss that the majority of the training programs aim to improve inspiratory muscle strength. However, they do not mention specific measures to improve endurance, and, when endurance is assessed, various techniques are used (19). MVV assessments provide more accurate information on respiratory muscle performance, and it can also be used as a method for respiratory muscle endurance training and improve the treatment of this population in the clinical setting (20).

The limitations of this study were: 1) the neurological level of spinal cord injury was not considered in the statistical analysis, and 2) the level of scoliosis was not described using Cobb angle. The MMC participants who had any walking ability were excluded because we could not measure the distance walked daily to understand their life style.

In conclusion, wheelchair-user MMC participants showed decreased vital capacity, as well as lower respiratory muscle endurance and increased weakness of the shoulder flexors and adductors, compared to healthy participants, suggesting lower ventilatory capacity and lower exercise endurance for this population.
